# Factors associated with anxiety among health care workers practicing in emergency department in south of tunisia

**DOI:** 10.1192/j.eurpsy.2021.494

**Published:** 2021-08-13

**Authors:** M. Tfifha, W. Abbes, I. Sellami, W. El Falah, M. Hajjaji, L. Ghanmi

**Affiliations:** 1 Department Of Psychiatry, regional hospital of gabes, gabes, Tunisia; 2 Occupational Medecine, university hospital Hédi Chaker-Sfax, Tunisia, Sfax, Tunisia

**Keywords:** mental health, stress management programs, anxiety disorder, health care workers

## Abstract

**Introduction:**

Health care workers in emergency department and intensive care are usually exposed to stressful situations, which require an early intervention.

**Objectives:**

To assess the prevalence of anxiety among health care workers in emergency department and to determine its associated factors.

**Methods:**

It was a cross-sectional, descriptive and analytical study including health care workers assigned to emergency ward and intensive care unit of Hedi Chaker and Habib Bourguiba hospitals in Sfax and the regional hospital of Kebili. Data was collected by an anonymous and confidential self- administered questionnaire. We used hospital anxiety and depression scale (HAD) to assess anxiety and depression.

**Results:**

The participation rate was 48.8% (n=240). The mean age was 37 years, 59.2% were female and 62% were married. Mean work experience was 11 years. 79.2% assured night shifts (average= 1.5 night shifts per week) and 71.7% benefited of compensatory rest. Our study revealed that 32.5 % of health care workers were suffering from anxiety. In univariate study, anxiety was significantly correlated with the female gender (p=0.004), the lack of practice of leisure activities (p=0.004), with absence of compensatory rest (p=0.001), with sleep disturbances (p=0.001) and with depression (p<10^-3^). Multivariate study pointed that anxiety was associated with lack of practice of leisure activities (OR=2.7[1.09-6.99]; p=0.006), absence of compensatory rest (OR=2.7[1.3-5.5]; p=0.004), depression (OR=3[1.5-6]; p= 0.001) and with sleep disturbances (OR=2.8[1.4-5.7]; p=0.004).

**Conclusions:**

Anxiety affected one in three emergency caregivers. Stress management programs for emergency caregivers can be of great help in dealing with this problem.

